# Pulmonary Interstitial Glycogenosis: An Unrecognized Etiology of Persistent Pulmonary Hypertension of the Newborn in Congenital Heart Disease?

**DOI:** 10.1007/s00246-012-0371-z

**Published:** 2012-05-22

**Authors:** Monique R. Radman, Patricia Goldhoff, Kirk D. Jones, Anthony Azakie, Sanjeev Datar, Ian Adatia, Peter E. Oishi, Jeffrey R. Fineman

**Affiliations:** 1 Department of Pediatrics, University of California, 513 Parnassus Avenue, Box 1346, San Francisco, CA 94143-1346 USA; 2 Cardiovascular Research Institute, University of California, San Francisco, CA 94143-1346 USA; 3 Department of Pathology, University of California, San Francisco, CA 94143-1346 USA; 4 Department of Surgery, University of California, San Francisco, CA 94143-1346 USA; 5 Department of Pediatrics, University of Alberta, Edmonton, AB Canada

**Keywords:** Congenital heart defects, Glycogen storage diseases, Interstitial lung diseases, Neonatal respiratory distress syndrome, Persistent pulmonary hypertension of the newborn

## Abstract

**Background:**

Pulmonary interstitial glycogenosis (PIG) arises from a developmental disorder of the pulmonary mesenchyme and presents clinically with reversible neonatal respiratory distress and/or persistent pulmonary hypertension of the newborn (PPHN).

**Objective:**

We report two cases of PIG in patients with congenital heart disease (CHD) and evidence of PPHN.

**Results:**

Both cases demonstrated the hallmark PIG histologic finding of diffuse, uniform interstitial thickening due to the presence of immature interstitial cells containing abundant cytoplasmic glycogen.

**Conclusions:**

We report the second and third patients with PIG associated with CHD. Because histologic examination is required to establish the diagnosis, we speculate that PIG, although rare, may be underrecognized in neonates presenting with PPHN in the setting of CHD.

## Introduction

Pediatric interstitial lung disease has an estimated prevalence of 1.3 to 3.6/1,000,000 [5, 6]. Although interstitial lung disease is a relatively uncommon cause of respiratory distress in the neonatal period, multiple types have been described. The majority of these types are genetic, inflammatory, or infectious in etiology. The diagnosis of interstitial lung disease is made based on the history, physical examination, imaging results, and, when deemed necessary and if feasible, results of more invasive diagnostic studies, including pulmonary function studies, bronchoalveolar lavage, and lung biopsy. Treatment of pediatric interstitial lung disease generally involves supportive care as well as anti-inflammatory medications [3].

Pulmonary interstitial glycogenosis (PIG), unlike most types of pediatric interstitial lung disease, arises from a developmental disorder. The etiology of the defect involves abnormal differentiation of the pulmonary mesenchyme. This abnormal differentiation results in the abundance of glycogen in pulmonary interstitial cells. The disorder most often presents in neonates with respiratory distress, with or without evidence of pulmonary hypertension, and diffuse interstitial infiltrates on chest radiograph. As with all interstitial lung diseases, the definitive diagnosis is made by histologic examination. For PIG, the hallmark histologic finding is diffuse, uniform interstitial thickening due to the presence of immature interstitial cells containing abundant cytoplasmic glycogen [7]. Treatment is supportive. Corticosteroid therapy is often effective. The efficacy of steroids is thought to relate to the acceleration of cellular maturation and not modulation of inflammation. The prognosis in PIG is more favorable than the majority of other pediatric interstitial lung diseases, but the reason for this remains unknown [1]. However, it may be fatal or associated with chronic lung disease.

We report two cases of PIG in patients with congenital heart disease (CHD) and clinical evidence of PPHN. To our knowledge, there is only one previous report of a patient with CHD and PIG in the literature. Unexplained PPHN can be associated with, and complicate the course of, neonates with CHD. Because histologic examination is required to establish the diagnosis, we speculate that PIG, a rare but potentially treatable form of PPHN, may be under-recognized in neonates presenting with PPHN in the setting of CHD.

## Case Report: Patient No. 1

This term, female infant was hypotonic and cyanotic shortly after an uncomplicated vaginal birth with saturations in the 70s on 100% oxygen masked with continuous positive airway pressure. The results of the initial arterial blood gas analysis were pH 7.25, partial pressure of carbon dioxide 58, partial pressure of oxygen 32, and base deficit −5. Umbilical catheters were placed; blood cultures were obtained; empiric antibiotic treatment was initiated; and transport was arranged to the UCSF neonatal intensive care unit (NICU). Initial as well as all subsequent blood cultures remained negative throughout the hospital course. Chest X-ray showed diffusely hazy lung fields with a normal cardiac silhouette. The patient was intubated due to persistent cyanosis and low oxygen saturations. Cyanotic CHD was suspected, and prostaglandin and dopamine continuous infusions were started to maintain ductal patency and adequate systemic blood pressure, respectively.

On arrival to the UCSF NICU, echocardiogram was obtained that showed d-transposition of the great arteries with normal coronary artery pattern, intact ventricular septum, patent foramen ovale with left-to-right shunting, large patent ductus arteriosus (PDA) with all left-to-right shunting, and normal ventricular function. The patient’s initial ICU course was remarkable for significant hemodynamic instability and hypoxemia despite positive-pressure mechanical ventilation, volume administration, and inotropic support with dopamine and epinephrine infusions. There was no response to inhaled nitric oxide. On hospital day 3, the patient was given a stress-dose of hydrocortisone in response to profound hypotension refractory to inotropes, which was concerning for cortisol deficiency. Marked clinical improvement occurred within 24 h of initiating steroid therapy. The epinephrine infusion was discontinued, and the dopamine infusion and inhaled nitric oxide were weaned. Two days later, the neonate required increasing inotropic support, and a second stress-dose of hydrocortisone was administered. Shortly after the initiation of the second steroid course, once again, her respiratory status improved dramatically. Inhaled nitric oxide was weaned, and the patient was successfully extubated. Subsequent serum adrenocorticotropic hormone and cortisol levels, drawn before the administration of steroids, returned to 10 and 15 μg/dL, respectively; both were within the range of normal, suggesting adequate adrenal function.

**Fig. 1:**
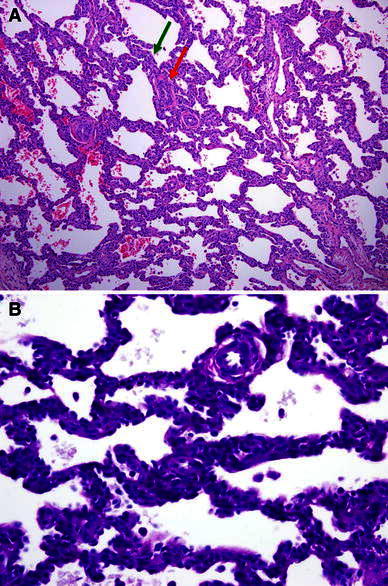
**a** A low-power view of the surgical lung biopsy specimen shows diffuse alveolar septal widening by interstitial cells. The pulmonary arteries surrounding the alveolar septum (*green arrow*) show moderate medial thickening (*red arrow*) (hematoxylin-and-eosin stain [×10]). **b** A high-power view shows granular cytoplasmic glycogen accumulation with the interstitial cells (PAS stain [×40])

On hospital day 13, the patient underwent arterial switch surgery, at which time an intraoperative lung biopsy specimen was performed. The surgical biopsy specimen showed patchy alveolar septal thickening by ovoid cells that were highlighted by periodic acid Schiff (PAS) stain, consistent with PIG [9, 11]. There was mild to moderate medial hypertrophy of the pulmonary arteries (Fig. 1).

The patient’s postoperative recovery was uncomplicated. Her pre-discharge echocardiogram showed intact arterial switch procedure results with mild tricuspid valve, pulmonary valve, and neo-aortic valve regurgitation, normal left-ventricular systolic function, and normal right-ventricular pressure and function. She was discharged home on hospital day 24.

## Case Report: Patient No. 2

This 37-day-old term infant boy presented shortly after birth with a murmur on cardiac auscultation and was initially diagnosed with tetralogy of Fallot. Shortly after birth, his saturations were in the mid 90s. However, during the subsequent 3 weeks, his oxygen saturations decreased to the low 70s. The parents did not report hypercyanotic spells, but he was tachypneic with feeds. His preadmission echocardiogram showed a mean transpulmonary gradient of 50 mmHg with a peak of 80 mmHg. He was admitted for observation and preoperative evaluation. Before surgery, he maintained saturations in the high 80s to low 90s with supplemental oxygen delivered by nasal cannula at a flow of ¼ L/min.

The patient underwent surgical repair on day of life 39, at which time preoperative transesophageal echocardiogram demonstrated heterotaxy syndrome (left atrial isomerism), double-outlet right ventricle, ventricular septal defect (VSD) with pulmonary stenosis with anomalous drainage of the right pulmonary veins to the right atrium, interrupted infrahepatic inferior vena cava with azygous continuation to the right superior vena cava, left superior vena cava that drained to the coronary sinus, and right-sided aortic arch with an aberrant left subclavian artery. He underwent surgical repair, which entailed a pulmonary arterioplasty to relieve the supravalvar stenosis, resection of the obstructing right-ventricular muscle bundles, closure of the VSD, division of the vascular ring, and baffling of the right partial anomalous pulmonary venous drainage to the left-sided atrium. Notably, before sternal closure, the distal pulmonary artery pressures and the right ventricular pressure were near systemic. Evaluation did not demonstrate any residual defects to account for these findings, and the pulmonary artery pressures were unresponsive to a trial of inhaled nitric oxide. Therefore, a lung biopsy specimen was obtained to investigate the etiology of the pulmonary arterial hypertension. The patient’s initial postoperative course was unremarkable, and his trachea was extubated on postoperative day 1. Due to persistent systemic right-ventricular pressures demonstrated on postoperative echocardiograms, therapy with sildenafil was initiated, and supplemental oxygen was administered to promote pulmonary vascular relaxation. The results of the intraoperative lung biopsy became available approximately 1 week after the surgery and showed PIG. Specifically, the sections contained diffuse alveolar septal thickening by numerous PAS-positive ovoid cells and minimal fibrosis. The pulmonary arteries were only minimally thickened. A paucity of T cells and B cells was noted.

Steroids were considered, but in contrast to patient no. 1, they were not given because the infant’s clinical course was one of gradual improvement with decreasing tachypnea. Subsequent echocardiogram demonstrated intact surgical repair results with no residual septal defects, mild to moderate tricuspid valve regurgitation, mild pulmonary valve regurgitation, and normal left-ventricular systolic function. Right-ventricular pressure was estimated at approximately 40 % systemic values. The infant was discharged on hospital day 39**,** with respiratory rates in the 40s to 50s, and oxygen saturations in the high 90s on supplemental oxygen by nasal cannula at ½ L/min flow.

## Discussion

We report, to the best of our knowledge, the second and third cases of PIG associated with CHD. Both patients presented with either clinical evidence (patient no. 1) or documentation (patient no. 2) of perioperative pulmonary hypertension that was clinically significant and unresponsive to inhaled nitric oxide. Interestingly, in patient no. 1, systemic steroid therapy was initiated before the diagnosis of PIG was made. Steroids were given for suspected, and subsequently disproved, adrenal insufficiency and were associated with clinical improvement. In patient no. 2, clinical improvement was gradual without steroid administration. Both cases demonstrated the hallmark PIG histologic finding of diffuse, uniform interstitial thickening due to the presence of immature interstitial cells containing abundant cytoplasmic glycogen. Moreover, both cases demonstrated evidence of increased pulmonary pressures, which led to the administration of inhaled nitric oxide and, in the second case, long-term treatment with sildenafil.

Persistent pulmonary hypertension of the newborn is not a single disease but rather a pathophysiologic condition. It is a clinical syndrome that manifests when the normal transition from the fetal to the postnatal pulmonary circulation fails to occur. In other words, the acronym “PPHN” encompasses the spectrum of diseases in which the normal sharp decrease in pulmonary vascular resistance and increase in pulmonary blood flow, with concomitant decrease in extrapulmonary shunting across the foramen ovale and DA, is significantly delayed [8]. The pathophysiologic mechanisms that prevent the normal pulmonary vascular remodeling and vasodilation at birth remain unclear, but they are most likely multifactorial in etiology. Several conditions, including sepsis, meconium aspiration syndrome, and lung hypoplasia, have been associated with PPHN [2, 10]. Not surprisingly, the response to therapy varies across etiologies and depends in part on the condition of the lung parenchyma and pulmonary vasculature. Moreover, some conditions that cause PPHN—such as alveolar capillary dysplasia (ACD) and pulmonary lymphangiectasias, particularly when it persists beyond the neonatal period as in seen patient no. 2—are almost entirely unresponsive to therapy. Thus, the ability to distinguish between the various conditions that result in PPHN to plan appropriate diagnostic studies, tailor therapies, and predict outcome remains crucial. It is recognized that PPHN may be an independent complicating factor in the preoperative and postoperative course of some neonates with CHD with severe hypoxemia and hemodynamic instability [12]. To date, potential etiologies for the independent association between PPHN and CHD have not been recognized.

Deutsch et al. [4] reported the first and only other documented case of PIG in a neonate with CHD. Similar to our cases, that neonate presented with severe pulmonary hypertension in the newborn period that could not be explained by the diagnosed CHD of hypoplastic aortic arch and PDA. Preoperative lung biopsy specimen demonstrated PIG, and systemic steroid therapy was initiated with marked clinical improvement. Interestingly, follow-up biopsy specimen at 2 months of age demonstrated resolution of the pathology.

We report two additional cases of PIG in neonates with CHD and evidence of unexplained pulmonary hypertension of the newborn. Because histologic examination is required to establish the diagnosis, we speculate that PIG, although rare, may be underrecognized in neonates presenting with PPHN in the setting of CHD. Moreover, although corticosteroids are the “gold-standard” therapy in biopsy-confirmed PIG, the consequences of this treatment, i.e., potential neurodevelopmental aberrations, immunosuppression, poor wound healing, and potential decreased pulmonary alveolarization in patients suffering from concomitant lung growth anomalies, must be considered because PIG is often a self-limited disease process*.*

## References

[1] Canakis A-M, Cutz E, Manson D (2002). Pulmonary interstitial glycogenosis: a new variant of neonatal interstitial lung disease. Am J Respir Crit Care Med.

[2] Clark RH, Kueser TJ, Walker MW, Southgate WM, Huckaby JL, Perez JA (2000). Low-dose nitric oxide therapy for persistent pulmonary hypertension of the newborn. N Engl J Med.

[3] Das S, Langston C, Fan LL (2011). Interstitial lung disease in children. Curr Opin Pediatr.

[4] Deutsch GH, Young LR (2009). Histologic resolution of pulmonary interstitial glycogenosis. Pediatr Dev Pathol.

[5] Dinwiddie R, Sharief N, Crawford O (2002). Idiopathic interstitial pneumonitis in children: a national survey in the United Kingdom and Ireland. Pediatr Pulmonol.

[6] Griese M, Haug M, Brasch F,  Freihorst A, Lohse P, von Kries R (2009). Incidence and classification of pediatric diffuse parenchymal lung diseases in Germany. Orphanet J Rare Dis.

[7] King BA, Boyd JT, Kingma PS (2011). Pulmonary maturational arrest and death in a patient with pulmonary interstitial glycogenosis. Pediatr Pulmonol.

[8] Konduri GG, Kim UO (2009). Advances in the diagnosis and management of persistent pulmonary hypertension of the newborn. Pediatr Clin North Am.

[9] Lanfranchi M, Allbery SM, Wheelock L, Perry D (2009). Pulmonary interstitial glycogenosis. Pediatr Radiol.

[10] Roberts JD, Fineman JR, Morin FC, Shaul PW, Rimar S, Schreiber MD (1997). Inhaled nitric oxide and persistent pulmonary hypertension of the newborn. N Engl J Med.

[11] Smets K, Daele S (2011). Neonatal pulmonary interstitial glycogenosis in a patient with Hunter syndrome. Eur J Pediatr.

[12] The Neonatal Inhaled Nitric Oxide Study Group (1997) Inhaled nitric oxide in full-term and nearly full-term infants with hypoxic respiratory failure. N Engl J Med 336:597–604 10.1056/NEJM199702273360901 9036320

